# Efficacy and safety of cabozantinib for patients with advanced hepatocellular carcinoma based on albumin-bilirubin grade

**DOI:** 10.1038/s41416-021-01532-5

**Published:** 2021-10-07

**Authors:** Robin Kate Kelley, Rebecca Miksad, Irfan Cicin, YenHsun Chen, Heinz-Josef Klümpen, Stefano Kim, Zhong-Zhe Lin, Jillian Youkstetter, Saswati Hazra, Suvajit Sen, Ann-Lii Cheng, Anthony B. El-Khoueiry, Tim Meyer, Ghassan K. Abou-Alfa

**Affiliations:** 1 UCSF Helen Diller Family Comprehensive Cancer Center, San Francisco, CA USA; 2 Beth Israel Deaconess Medical Center, Boston, MA USA; 3 Trakya University, Edirne, Turkey; 4 The Chinese University of Hong Kong, Hong Kong, China; 5 Amsterdam University Medical Centers, University of Amsterdam, Amsterdam, The Netherlands; 6 Centre Hospitalier Régional Universitaire de Besançon, Besançon, France; 7 National Taiwan University Hospital, Taipei, Taiwan; 8 Exelixis, Inc, Alameda, CA USA; 9 USC Norris Comprehensive Cancer Center, Los Angeles, CA USA; 10 Royal Free Hospital, University College London, London, UK; 11 Memorial Sloan Kettering Cancer Center, New York, NY USA; 12 Weill Medical College at Cornell University, New York, NY USA; 13 Present Address: Flatiron Health, Inc, New York, NY USA; 14 Present Address: Boston Medical Center, Boston, MA USA

**Keywords:** Drug development, Hepatocellular carcinoma

## Abstract

**Background:**

Albumin-bilirubin (ALBI) grade is an objective measure of liver function for patients with hepatocellular carcinoma (HCC). The tyrosine kinase inhibitor cabozantinib is approved for patients with advanced HCC who have received prior sorafenib based on the phase 3 CELESTIAL trial (NCT01908426). Cabozantinib improved overall survival (OS) and progression-free survival (PFS) versus placebo in patients with previously treated HCC.

**Methods:**

Patients were randomised 2:1 to receive cabozantinib 60 mg or placebo orally every day. Clinical outcomes in patients with ALBI grade 1 or 2 at baseline were evaluated in CELESTIAL. ALBI scores were retrospectively calculated based on baseline serum albumin and total bilirubin, with an ALBI grade of 1 defined as  ≤ −2.60 score and a grade of 2 as a score of > −2.60 to  ≤ −1.39.

**Results:**

Cabozantinib improved OS and PFS versus placebo in both ALBI grade 1 (hazard ratio [HR] [95% CI]: 0.63 [0.46–0.86] and 0.42 [0.32–0.56]) and ALBI grade 2 (HR [95% CI]: 0.84 [0.66–1.06] and 0.46 [0.37–0.58]) subgroups. Adverse events were consistent with those in the overall population. Rates of grade 3/4 adverse events associated with hepatic decompensation were generally low and were more common among patients in the ALBI grade 2 subgroup.

**Discussion:**

These results provide initial support of cabozantinib in patients with advanced HCC irrespective of ALBI grade 1 or 2.

**Trial registration number:**

ClinicalTrials.gov number, NCT01908426.

## Background

Patients with hepatocellular carcinoma (HCC) frequently present with underlying cirrhosis, the severity of which is generally assessed by the Child-Pugh grade [1, 2]. Most clinical trials of systemic therapies in HCC are limited to patients with Child-Pugh grade A liver cirrhosis, including the phase 3 CELESTIAL trial of cabozantinib in HCC [1–3]. However, heterogeneity exists among Child-Pugh grade A patients, attributed in part to the requirement for clinical assessments of ascites and encephalopathy, which introduces subjectivity [4, 5]. To provide a more objective measure of liver function, a scoring system based on serum albumin and bilirubin (ALBI) was developed based on laboratory data from a large international cohort of patients [4, 5]. Within the Child-Pugh A category, patients can be furthered categorised by ALBI score, most often corresponding to ALBI grade 1 or ALBI grade 2; with higher ALBI grades associated with worse liver dysfunction and overall poor prognosis [4, 5].

In the phase 3 CELESTIAL trial, cabozantinib, a tyrosine kinase inhibitor, which inhibits MET, VEGFR and AXL, significantly improved overall survival (OS) and progression-free survival (PFS) versus placebo in patients with previously treated advanced HCC [3, 6]. For the overall CELESTIAL population, median OS was 10.2 months with cabozantinib versus 8.0 months with placebo (hazard ratio [HR] 0.76; 95% confidence interval [CI] 0.63–0.92; *p* = 0.005), and median PFS was 5.2 months with cabozantinib versus 1.9 months with placebo (HR 0.44; 95% CI 0.36–0.52; *p* < 0.001) [3]. Considering the limitations of Child-Pugh scoring, and to better delineate the potential impact of liver function on treatment outcomes in CELESTIAL, we assessed key outcomes based on ALBI grade at study baseline.

## Methods

CELESTIAL study details have been previously published [3]. Briefly, adult patients with advanced HCC, Child-Pugh grade A liver function, and Eastern Cooperative Oncology Group (ECOG) performance status (PS) of 0 or 1 were eligible. Patients must have received prior sorafenib and could have received up to two prior systemic regimens for HCC. Patients were randomised 2:1 to receive cabozantinib 60 mg or placebo orally every day. The primary outcome was OS and key secondary efficacy outcomes were PFS and objective response rate (ORR) by investigator per RECIST v1.1. Adverse events were reported according to National Cancer Institute Common Terminology Criteria for Adverse Events v4.0 [7]. Serum albumin and total bilirubin were measured centrally at study baseline (within 7 days prior to randomisation) [3] and used to retrospectively calculate ALBI score: (log_10_ bilirubin µmol/L × 0.66) + (albumin g/L × −0.085) [4]. An ALBI grade of 1 was defined as ≤−2.60 score, while a grade of 2 was a score of >−2.60 to ≤−1.39 [4]. A post-hoc multivariable analysis was performed to evaluate the association of ALBI grade and other baseline variables with OS. The same Cox proportional hazard model was run independently for each treatment arm with the following variables: ALBI grade (2 vs. 1), alpha fetoprotein (AFP) (≥400 vs. <400 ng/mL), ECOG PS (≥1 vs 0), macrovascular invasion (MVI, yes vs. no), extrahepatic spread (yes vs. no), age (<65 vs. ≥65 years), gender and aetiology (hepatitis B virus, hepatitis C virus, other). Hazard ratio, 95% CI and *p*-values were determined.

## Results

Among 707 patients who were randomised 2:1 to receive cabozantinib (60 mg daily) or placebo, 186 patients (40%) had ALBI score grade 1 and 282 patients (60%) had ALBI score grade 2 in the cabozantinib arm. One hundred and two patients (43%) had ALBI score grade 1 and 133 patients (57%) had ALBI score grade 2 in the placebo arm. Two patients in each treatment arm had ALBI grade 3 and were not included in this analysis. In the ALBI grade 1 subgroup, 60% of patients in the cabozantinib arm versus 64% in the placebo arm had an ECOG PS of 0, while 40% of patients in the cabozantinib arm versus 36% in the placebo arm had an ECOG PS of 1 (Table 1). In the ALBI grade 2 subgroup, 47% patients in the cabozantinib arm versus 49% in the placebo arm had an ECOG PS of 0 while 52% patients in the cabozantinib arm versus 51% in the placebo arm had an ECOG PS of 1. Hepatitis C was present in 17% of patients in the cabozantinib arm versus 15% in the placebo arm in the ALBI grade 1 subgroup and 28% of patients in the cabozantinib arm versus 29% in the placebo arm in the ALBI grade 2 subgroup. Twenty patients from the ALBI grade 2 subgroup had ascites versus nine patients in the ALBI grade 1 subgroup and three patients in the ALBI grade 2 subgroup had Grade I–II encephalopathy with none in the ALBI grade 1 subgroup. The median duration of prior sorafenib treatment was 4.4 months for patients in both treatment arms in the ALBI grade 1 subgroup, while it was 5.8 months in the cabozantinib arm versus 6.1 months in the placebo arm in the ALBI grade 2 subgroup. In the ALBI grade 1 subgroup, 94% patients in the cabozantinib arm versus 97% in the placebo arm had a Child-Pugh A5 score while 5% patients in the cabozantinib arm versus 3% in the placebo arm had a Child-Pugh A6 score. In the ALBI grade 2 subgroup, 32% patients in the cabozantinib arm versus 41% in the placebo arm had a Child-Pugh A5 score, while 61% patients in the cabozantinib arm versus 56% in the placebo arm had a Child-Pugh A6 score. Baseline characteristics were generally balanced between treatment arms within each of these ALBI grade subgroups. The Chi-Square *p*-value to test the homogeneity of ALBI score grade 1 versus score grade 2 subgroups was >0.05 thereby concluding that these 2 subgroups were not heterogenous.

**Table 1:** Baseline demographics and clinical characteristics by ALBI grade.

	ALBI grade 1			ALBI grade 2		
	Cabozantinib (*N* = 186)	Placebo (*N* = 102)	Total (*N* = 288)	Cabozantinib (*N* = 282)	Placebo (*N* = 133)	Total (*N* = 415)
Age, median (range), years	62.0 (28–85)	63.5 (34–86)	63.0 (28–86)	65.0 (22–86)	65.0 (24–85)	65.0 (22–86)
Male, *n* (%)	143 (77)	87 (85)	230 (80)	234 (83)	113 (85)	347 (84)
Geographic regions^a^, *n* (%)						
Asia	53 (28)	29 (28)	82 (28)	63 (22)	30 (23)	93 (22)
Europe	86 (46)	50 (49)	136 (47)	144 (51)	57 (43)	201 (48)
Pacific	4 (2)	6 (6)	10 (3)	11 (4)	5 (4)	16 (4)
North America	43 (23)	17 (17)	60 (21)	64 (23)	41 (31)	105 (25)
Race, *n* (%)						
Asian	73 (39)	44 (43)	117 (41)	86 (30)	38 (29)	124 (30)
White	96 (52)	46 (45)	142 (49)	168 (60)	82 (62)	250 (60)
Black	3 (2)	3 (3)	6 (2)	4 (1)	8 (6)	12 (3)
Other	2 (1)	0	2 (1)	5 (2)	2 (2)	7 (2)
Not reported	12 (6)	9 (9)	21 (7)	19 (7)	3 (2)	22 (5)
ECOG status, *n* (%)						
0	111 (60)	65 (64)	176 (61)	133 (47)	65 (49)	198 (48)
1	75 (40)	37 (36)	112 (39)	146 (52)	68 (51)	216 (52)
Etiology of disease^b^, *n* (%)						
HBV	78 (42)	39 (38)	117 (41)	99 (35)	49 (37)	148 (36)
HCV	32 (17)	15 (15)	47 (16)	80 (28)	38 (29)	118 (28)
Dual HBV and HCV infection	1 (1)	0	1 (<1)	7 (2)	3 (2)	10 (2)
Alcohol	30 (16)	18 (18)	48 (17)	81 (29)	21 (16)	102 (25)
Nonalcoholic steatohepatitis	15 (8)	11 (11)	26 (9)	28 (10)	12 (9)	40 (10)
Albumin, *n* (%)						
<35 g/L	0	0	0	129 (46)	58 (44)	187 (45)
≥35 g/L	186 (100)	102 (100)	288 (100)	153 (54)	75 (56)	228 (55)
Bilirubin, *n* (%)						
<22.23 µmol/L	183 (98)	102 (100)	285 (99)	237 (84)	118 (89)	355 (86)
≥22.23–<29.07 µmol/L	3 (2)	0	3 (1)	34 (12)	12 (9)	46 (11)
≥29.07 µmol/L	0	0	0	11 (4)	3 (2)	14 (3)
AFP, *n* (%)						
<400 ng/mL	115 (62)	63 (62)	178 (62)	162 (57)	72 (54)	234 (56)
≥400 ng/mL	71 (38)	39 (38)	110 (38)	120 (43)	61 (46)	181 (44)
Extrahepatic spread of disease and/or macrovascular invasion, *n* (%)	164 (88)	84 (82)	248 (86)	232 (82)	114 (86)	346 (83)
Extrahepatic spread of disease	157 (84)	78 (76)	235 (82)	210 (74)	102 (77)	312 (75)
Macrovascular invasion	35 (19)	23 (23)	58 (20)	93 (33)	58 (44)	151 (36)
Child-Pugh, *n* (%)						
A^c^	186 (100)	102 (100)	288 (100)	274 (97)	131 (98)	405 (98)
A5	174 (94)	99 (97)	273 (95)	90 (32)	54 (41)	144 (35)
A6	9 (5)	3 (3)	12 (4)	173 (61)	75 (56)	248 (60)
B	0	0	0	7 (2)	2 (2)	9 (2)
Sites of disease, *n* (%)						
Liver	138 (74)	89 (87)	227 (79)	255 (90)	125 (94)	380 (92)
Bone	22 (12)	14 (14)	36 (13)	38 (13)	20 (15)	58 (14)
Visceral (excluding liver)	90 (48)	46 (45)	136 (47)	123 (44)	58 (44)	181 (44)
Lymph node	54 (29)	28 (27)	82 (28)	100 (35)	43 (32)	143 (34)
Number of prior systemic anticancer regimens for advanced HCC, *n* (%)^d^						
1	132 (71)	71 (70)	203 (70)	202 (72)	101 (76)	303 (73)
2	52 (28)	31 (30)	83 (29)	77 (27)	31 (23)	108 (26)
Chemoembolisation for HCC, *n* (%)	83 (45)	42 (41)	125 (43)	120 (43)	69 (52)	189 (46)
Median total duration of prior sorafenib (range), mo	4.4 (0.4–40.5)	4.4 (0.3–42.9)	4.4 (0.3–42.9)	5.8 (0.3–70.0)	6.1 (0.2–76.8)	5.8 (0.2–76.8)
Median time from disease progression to randomisation (range), mo	1.6 (0.1–42.3)	1.6 (0.3–14.5)	1.6 (0.1–42.3)	1.6 (0.0–100.8)	1.7 (0.2–69.4)	1.6 (0.0–100.8)

*AFP* alpha fetoprotein, *ALBI* albumin–bilirubin, *ECOG* Eastern Cooperative Oncology Group, *HBV* hepatitis B virus, *HCC* hepatocellular carcinoma, *HCV* hepatitis C virus, *mo* months.

^a^Asia includes Republic of Korea, Hong Kong, Taiwan and Singapore. Pacific includes Australia and New Zealand.

^b^Etiology per case report form (some patients had >1 disease etiology category).

^c^Includes six patients who were given an A grade with no score and 10 patients given an A grade with a 7 score.

^d^Two patients in cabozantinib arm and one patient in the placebo arm received ≥3 prior lines of therapy; all patients were ALBI grade 2 at baseline.

**Fig. 1:**
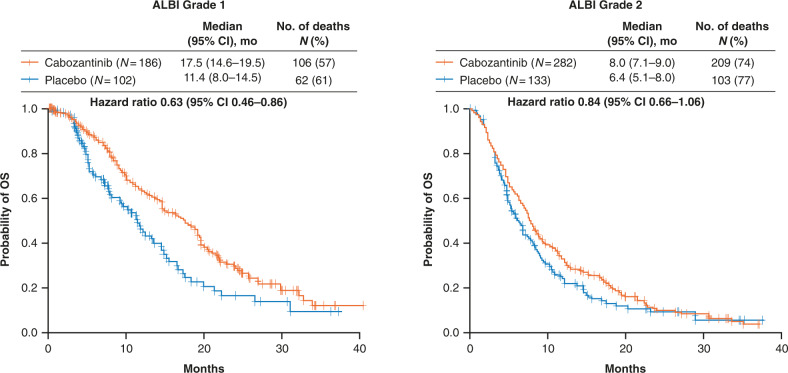
Overall survival by ALBI grade. Hazard ratios are unstratified. ALBI albumin–bilirubin, CI confidence interval, OS overall survival.

Within the limitations of this being a retrospective evaluation with relatively small sample sizes of individual subgroups, the OS was longer for patients receiving cabozantinib versus placebo for the ALBI grade 1 subgroup (HR 0.63, 95% CI 0.46–0.86), while for the ALBI grade 2 subgroup, HR was 0.84 (95% CI 0.66–1.06) (Fig. 1). The median OS was 17.5 (95% CI 14.6–19.5) months in the cabozantinib arm versus 11.4 months (95% CI 8.0–14.5) in the placebo arm for the ALBI grade 1 subgroup, and 8.0 months (95% CI 7.1–9.0) in the cabozantinib arm versus 6.4 months (95% CI 5.1–8.0) in the placebo arm for the ALBI grade 2 subgroup. In a multivariable analysis, baseline ALBI grade 2 was independently associated with reduced OS compared with ALBI grade 1 in both the cabozantinib (HR 1.99, *p* < 0.0001) and placebo (HR 1.67, *p* = 0.0021) arms (Table 2). A greater percentage of patients with ALBI grade 1 received subsequent anticancer therapy compared with those with ALBI grade 2, with rates similar between the treatment arms (Supplemental Table S1).

**Table 2:** Multivariable analysis of overall survival.

	Cabozantinib arm		Placebo arm	
	*p* value	Hazard ratio	*p* value	Hazard ratio
ALBI grade, 2 vs. 1	<0.0001	1.99	0.0021	1.67
MVI, yes vs. no	<0.0001	1.65	0.0019	1.73
EHS, yes vs. no	0.0492	1.32	0.0150	1.65
ECOG PS, ≥1 vs. 0	0.0009	1.47	0.7677	1.05
AFP, ≥400 vs. <400 ng/mL	<0.0001	1.71	0.0008	1.75

*AFP* alpha fetoprotein, *ALBI* albumin–bilirubin, *ECOG* Eastern Cooperative Oncology Group, *EHS* extrahepatic spread, *MVI* macrovascular invasion, *PS* performance status.

**Fig. 2:**
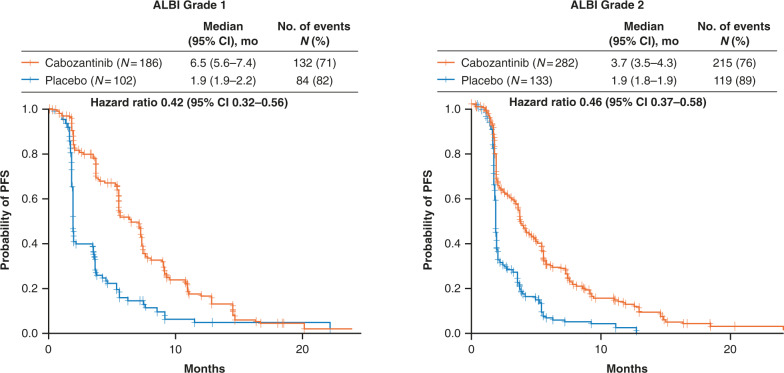
Progression-free survival by ALBI grade. Hazard ratios are unstratified. ALBI, albumin–bilirubin, CI confidence interval, PFS progression-free survival.

Progression-free survival with cabozantinib was longer than placebo for the ALBI grade 1 (HR 0.42, 95% CI 0.32–0.56) and ALBI grade 2 (HR 0.46, 95% CI 0.37–0.58) subgroups (Fig. 2). In patients with ALBI grade 1, median PFS was 6.5 months (95% CI 5.6–7.4) with cabozantinib versus 1.9 months (95% CI 1.9–2.2) with placebo, while in patients with ALBI grade 2, median PFS was 3.7 months (95% CI 3.5–4.3) with cabozantinib versus 1.9 months (95% CI 1.8 − 1.9) with placebo. The ORR was 4% in the cabozantinib arm for both the subgroups, versus 1% in the placebo arm for the ALBI grade 1 subgroup and 0% in the ALBI grade 2 subgroup (Supplemental Table S2). The disease control rate (complete/partial responses + stable disease) was greater with cabozantinib versus placebo in both ALBI subgroups as well; for the ALBI grade 1 subgroup, the disease control rate was 74% in the cabozantinib arm versus 40% in the placebo arm; for the ALBI grade 2 subgroup, the disease control rate was 57% in the cabozantinib arm versus 28% in the placebo arm.

The median average daily dose of cabozantinib was 36.7 mg in the ALBI grade 1 subgroup and 35.3 mg in the ALBI grade 2 subgroup while the median duration of exposure was 4.9 months in the ALBI grade 1 subgroup and 3.3 months in the ALBI grade 2 subgroup (Table 3). In the ALBI grade 1 subgroup, 12% of patients discontinued the study due to treatment-related adverse events (AEs) in the cabozantinib arm versus 2% in the placebo arm. In the ALBI grade 2 subgroup, 19% of patients discontinued due to treatment-related AEs in the cabozantinib arm versus 4% in the placebo arm. Seventy-five percent of patients treated with cabozantinib experienced grade 3/4 AEs in the ALBI grade 1 subgroup while 63% experienced grade 3/4 AEs in the ALBI grade 2 subgroup (Table 4). Hypertension and palmar-plantar erythrodysesthesia were the most common grade 3/4 AEs in both the subgroups. Grade 3/4 AEs associated with hepatic decompensation included ascites and hepatic encephalopathy. Rates for grade 3/4 ascites were 0.5% (*n* = 1) for cabozantinib versus 1.0% (*n* = 1) for placebo in the ALBI grade 1 subgroup, and 6.1% (*n* = 17) versus 7.5% (*n* = 10) in the ALBI grade 2 subgroup, respectively. Rates for grade 3/4 hepatic encephalopathy were 0.5% (*n* = 1) for cabozantinib versus 0% for placebo in the ALBI grade 1 subgroup, and 4.3% (*n* = 12) versus 1.5% (*n* = 2) in the ALBI grade 2 subgroup.

**Table 3:** Study treatment exposure, dose reduction and discontinuations by ALBI grade (safety population).

	ALBI grade 1		ALBI grade 2	
	Cabozantinib (*N* = 186)	Placebo (*N* = 102)	Cabozantinib (*N* = 279)	Placeb (*N* = 133)
Median duration of exposure (range), months	4.9 (0.1–31.8)	2.1 (0.1–27.2)	3.3 (0.1–37.3)	2.0 (0.0–13.5)
Median average daily dose (range), mg	36.7 (1.1–60.0)	58.9 (21.8–60.0)	35.3 (3.9–60.0)	58.7 (12.0–60.0)
Discontinuation due to treatment-related adverse event, n (%)	23 (12)	2 (2)	52 (19)	5 (4)

*ALBI* albumin–bilirubin.

**Table 4:** All-causality grade 3 or 4 adverse events by ALBI grade (safety population)^a^.

	ALBI grade 1		ALBI grade 2	
	Cabozantinib (*N* = 186)	Placebo (*N* = 102)	Cabozantinib (*N* = 279)	Placebo (*N* = 133)
Any grade 3 or 4 adverse event, *n* (%)	140 (75)	35 (34)	176 (63)	50 (38)
Hypertension	40 (22)	3 (3)	34 (12)	1 (1)
PPE	33 (18)	0	46 (16)	0
Diarrhea	25 (13)	1 (1)	21 (8)	3 (2)
AST increased	15 (8)	4 (4)	40 (14)	11 (8)
Fatigue	14 (8)	2 (2)	35 (13)	8 (6)
Decreased appetite	9 (5)	0	18 (6)	1 (1)
Asthenia	8 (4)	2 (2)	24 (9)	2 (2)
Anemia	5 (3)	1 (1)	14 (5)	11 (8)

*ALBI* albumin–bilirubin, *AST* aspartate aminotransferase, *PPE* palmar-plantar erythrodysesthesia.

^a^Events that occurred at ≥5.0% frequency in either treatment arm in the overall safety population are summarised.

## Discussion

In this retrospective analysis, we evaluated treatment outcomes from CELESTIAL based on baseline ALBI grade to better define the potential impact of liver function on outcomes with cabozantinib. At baseline, patients with ALBI grade 1 were more likely to have better ECOG PS and less likely to have hepatitis C virus and MVI compared to those with ALBI grade 2. A greater percentage of patients had a Child-Pugh A5 score and a lower percentage had a Child-Pugh A6 score in the ALBI grade 1 subgroup compared to the ALBI grade 2 subgroup (95% vs. 35% and 4% vs. 60%, respectively).

Patients with ALBI grade 1 treated with cabozantinib had longer OS and PFS compared with patients receiving placebo. Patients with ALBI grade 2 also achieved a significant prolongation of PFS with cabozantinib along with a trend towards longer OS, although not reaching statistical significance. In the multivariable analysis, baseline ALBI grade 1 was independently associated with improved OS for both treatment arms. The ORR reported in the overall study results (4% for the cabozantinib and ≤1% for placebo) were generally maintained in both subgroups by treatment [3]. Overall, the efficacy findings reported by ALBI subgroup were consistent with overall results from CELESTIAL, although higher ALBI grade was a negative prognostic factor in both treatment arms. The significant improvements in the secondary efficacy outcomes of PFS and disease control rate coupled with the OS improvements suggest cabozantinib improves treatment outcomes across the spectrum of Child-Pugh A and ALBI grades 1 and 2 liver function. These results are reinforced by the finding that cabozantinib also improved outcomes over placebo in patients enrolled in CELESTIAL whose liver function had deteriorated to Child-Pugh B by Week 8 on treatment, supporting the efficacy of cabozantinib in HCC across a range of liver dysfunction [8].

The ALBI grade 2 subgroup was associated with a higher frequency of the liver decompensation events of ascites and encephalopathy compared to the ALBI grade 1 subgroup. The overall higher incidence of grade 3/4 AEs with cabozantinib for the ALBI grade 1 versus 2 subgroups may have been in part due to differences in the median duration of exposure (ALBI grade 1: 4.9 months, ALBI grade 2: 3.3 months). Generally the most common grade 3/4 adverse AEs in both subgroups were consistent with those in the overall population [3]. In the subgroup analysis based on liver function in patients from CELESTIAL whose cirrhosis evolved to Child-Pugh B by Week 8, cabozantinib treatment had a similar safety profile [8]. This suggests that cabozantinib maintained a consistent safety profile in CELESTIAL irrespective of the status of liver function.

In real world studies, nearly all patients with ALBI grade 1 had Child-Pugh A, while <1% had Child-Pugh B; for ALBI grade 2, the majority of patients had Child-Pugh A, but 22–27% had Child-Pugh B [9, 10]. In one of these studies, a large, retrospective analysis of 1019 patients treated with sorafenib for HCC across 17 European centers, over 90% of patients with ALBI grade 1 also had Child-Pugh score A5, while those with ALBI grade 2 had higher proportion with Child-Pugh A6 [9]. These findings were similar to the findings from this subgroup analysis of patients enrolled in the CELESTIAL trial of patients treated as a second or third line of therapy, which may be associated with poorer prognosis overall than a first-line treatment context. Future analyses are warranted to compare the relative prognostic value for Child-Pugh scores of A5 versus A6 with ALBI grades 1 versus 2 in prospective clinical trial populations with clinical annotation for presence of ascites and encephalopathy as well as ECOG score, subjective factors which may be missing from retrospective databases and which may not be captured consistently in clinical practice outside of clinical trials. These analyses could support use of ALBI grade as an objective stratification factor in future clinical trials. For clinical practice, the more accessible EZ ALBI score, which uses a more simplified formula than ALBI grade, could potentially be used, if prospective studies support this approach [11].

## Conclusions

In summary, cabozantinib improved clinical outcomes compared with placebo in patients with advanced HCC and the benefits associated with cabozantinib were symmetric in the subgroups defined by ALBI grade 1 or 2. Although this analysis was post-hoc and hypothesis generating, ALBI grade demonstrated good prognostic discrimination and may be considered for subgroup analyses and potential stratification in future randomised control trials.

### Reporting summary

Further information on research design is available in the Nature Research Reporting Summary linked to this article.

## Supplementary information

Supplemental Material

Reporting Summary Checklist

## Data Availability

All data relevant to the study are included in the article or uploaded as supplementary information.
