# Supplementary material for: Efficacy and safety of cabozantinib for patients with advanced hepatocellular carcinoma based on albumin-bilirubin grade
Source: Br J Cancer. 2021 Oct 7;126(4):569–75. doi: 10.1038/s41416-021-01532-5 (PMC8854685; doi:10.1038/s41416-021-01532-5)
Supplement: Supplementary file 1 — Supplemental Material [file 41416_2021_1532_MOESM1_ESM.docx]

**Supplemental material**

**Table S1.** Subsequent anticancer therapy by ALBI grade^a^

|  | **ALBI grade 1** | | **ALBI grade 2** | |
| --- | --- | --- | --- | --- |
|  | **Cabozantinib**  **(N = 186)** | **Placebo**  **(N = 102)** | **Cabozantinib (N = 282)** | **Placebo**  **(N = 133)** |
| Any TKI, n (%) | 23 (12) | 5 (5) | 11 (4) | 3 (2) |
| Sorafenib | 13 (7) | 1 (1) | 6 (2) | 3 (2) |
| Regorafenib | 7 (4) | 3 (3) | 4 (1) | 0 |
| Anti–PD-1/PD-L1, n (%) | 14 (8) | 11 (11) | 8 (3) | 4 (3) |
| Cytotoxic chemotherapy, n (%) | 30 (16) | 22 (22) | 27 (10) | 17 (13) |

^a^Patients could have received combination regimens that included more than one agent

ALBI, albumin-bilirubin; PD-1, programmed cell death-1; PD-L1, programmed death-ligand 1; TKI, tyrosine kinase inhibitor

**Table S2.** Best overall tumour response by ALBI grade

|  | **ALBI grade 1** | | **ALBI grade 2** | |
| --- | --- | --- | --- | --- |
|  | **Cabozantinib**  **(N = 186)** | **Placebo**  **(N = 102)** | **Cabozantinib**  **(N = 282)** | **Placebo**  **(N = 133)** |
| Objective response rate,^a^ (95% CI), % | 4 (1.9–8.3) | 1 (0–5.3) | 4 (1.7–6.4) | 0 (0–0) |
| Best overall response, n (%) |  |  |  |  |
| Partial response | 8 (4) | 1 (1) | 10 (4) | 0 |
| Stable disease | 129 (69) | 40 (39) | 152 (54) | 37 (28) |
| Progressive disease | 28 (15) | 54 (53) | 69 (24) | 77 (58) |
| Disease control rate, (95% CI), % | 74 (67–80) | 40 (31–50) | 57 (51–63) | 28 (20–36) |

^a^All responses were partial responses. Disease control rate=complete response+partial response+stable disease

ALBI, albumin-bilirubin; CI, confidence interval
